# Incorporating Uncertainty Estimation and Interpretability in Personalized Glucose Prediction Using the Temporal Fusion Transformer

**DOI:** 10.3390/s25154647

**Published:** 2025-07-26

**Authors:** Antonio J. Rodriguez-Almeida, Carmelo Betancort, Ana M. Wägner, Gustavo M. Callico, Himar Fabelo, on behalf of the WARIFA Consortium

**Affiliations:** 1 Institute for Applied Microelectronics, University of Las Palmas de Gran Canaria, ULPGC, 35017 Las Palmas de Gran Canaria, Spain; gustavo@iuma.ulpgc.es; 2 Endocrinology and Nutrition Department, Complejo Hospitalario Universitario Insular Materno-Infantil, CHUIMI, 35016 Las Palmas de Gran Canaria, Spain; carmelo.betancort@fpct.ulpgc.es (C.B.); ana.wagner@ulpgc.es (A.M.W.); 3 Instituto de Investigaciones Biomédicas y Sanitarias, University of de Las Palmas de Gran Canaria, ULPGC, 35016 Las Palmas de Gran Canaria, Spain; 4 Fundación Canaria Instituto de Investigación Sanitaria de Canarias, FIISC, 35019 Las Palmas de Gran Canaria, Spain; 5 Research Unit, Hospital Universitario de Gran Canaria Dr. Negrin, 35010 Las Palmas de Gran Canaria, Spain

**Keywords:** glucose prediction, transformers, artificial intelligence, explainable AI, deep learning, personalized medicine, mHealth

## Abstract

More than 14% of the world’s population suffered from diabetes mellitus in 2022. This metabolic condition is defined by increased blood glucose concentrations. Among the different types of diabetes, type 1 diabetes, caused by a lack of insulin secretion, is particularly challenging to treat. In this regard, automatic glucose level estimation implements Continuous Glucose Monitoring (CGM) devices, showing positive therapeutic outcomes. AI-based glucose prediction has commonly followed a deterministic approach, usually with a lack of interpretability. Therefore, these AI-based methods do not provide enough information in critical decision-making scenarios, like in the medical field. This work intends to provide accurate, interpretable, and personalized glucose prediction using the Temporal Fusion Transformer (TFT), and also includes an uncertainty estimation. The TFT was trained using two databases, an in-house-collected dataset and the OhioT1DM dataset, commonly used for glucose forecasting benchmarking. For both datasets, the set of input features to train the model was varied to assess their impact on model interpretability and prediction performance. Models were evaluated using common prediction metrics, diabetes-specific metrics, uncertainty estimation, and interpretability of the model, including feature importance and attention. The obtained results showed that TFT outperforms existing methods in terms of RMSE by at least 13% for both datasets.

## 1. Introduction

In 2022, around 830 million people were affected by diabetes mellitus (DM), accounting for more than 14% of adults worldwide [1]. This chronic metabolic condition is defined by increased blood glucose concentrations [2,3], which are associated with health complications, such as damage to the kidneys, blood vessels, eyes, or heart, negatively impacting the well-being of those affected [1]. There are two main types of DM: type 2 diabetes, produced by increased resistance to insulin and the inability of the body to secrete enough insulin to overcome such resistance, and type 1 diabetes (T1D), caused by an autoimmune, pancreatic beta-cell destruction that leads to a complete lack of insulin production [1,4]. T1D treatment is particularly challenging, since suboptimal, exogenous insulin administration is needed to keep blood glucose within a safe range, avoiding hypo- and hyperglycemic events (low and high blood glucose levels, respectively) [5,6]. Subsequently, glycemic control in people with T1D demands a lifetime of exhaustive self-management. To achieve good glycemic control and minimize the occurrence of harmful hyper- and hypoglycemic events, accurate daily insulin administration is essential for people with T1D [1,7].

Among the available tools for T1D self-management, Continuous Glucose Monitoring (CGM) has become a standard of care in many countries [8,9]. CGM devices consist of a sensor inserted under the individual’s skin that tracks interstitial glucose concentrations (in this work called CGM readings) every 1 to 15 min, depending on the sensor, and a transmitter, which sends the information further to a monitor, watch, phone, or pump [10]. The use of CGM has helped to reduce the number of hypoglycemic events in people with T1D [11], also enabling the incorporation of glucose prediction into insulin delivery automation through so-called hybrid closed-loop systems. The latter consists of an insulin pump, a CGM device with an incorporated transmitter, and a controller algorithm that uses the information from the CGM to adjust insulin delivery rates [10]. In this work, CGM data refers to all the information provided by the CGM sensor (i.e., CGM readings and timestamps), whereas CGM readings refer only to the interstitial glucose values measured by the device.

Glucose prediction is already included in the hybrid closed-loop systems to guide automatic insulin delivery [12]. In this regard, artificial intelligence (AI)-based glucose level forecasting has been an active area of research in recent years. A significant number of studies are based on Convolutional Neural Networks (CNNs) [13,14,15,16,17] and Long Short-Term Memories (LSTMs) [16,18]. However, although there are post hoc approaches to interpret these models (e.g., Shapley Additive exPlanations (SHAP) [19]), these approaches present two main drawbacks which are especially relevant in glucose prediction: the *black-box* nature of their architecture and the lack of information inherent to the deterministic prediction (i.e., predicting a single point rather than a range within which the prediction could fall). The former is critical to understand the *reasoning* underlying an AI-based decision that involves human lives [20]. The latter strongly limits the information provided to the end-user (i.e., a person with T1D or a clinician), who is unaware of the *uncertainty* of the provided prediction [21].

In data modeling, two types of uncertainties can be found. Firstly, *aleatoric uncertainty*, which is inherent to the random nature of the studied phenomenon (in this case, glucose level variations over time). This uncertainty cannot be reduced, but it can be identified and estimated. Secondly, *epistemic uncertainty* (also known as *model uncertainty*) is associated with the model’s lack of knowledge of the studied data. This uncertainty can be reduced with effective model training and a suitable architecture [22]. In this regard, Gal and Ghahramani achieved a significant milestone in 2016. They demonstrated that the use of drop-out layers during the test phase in deep learning (DL) models enables model (epistemic) uncertainty estimation. This technique was called Monte-Carlo Drop-Out (MCDO) [23]. Nonetheless, the main disadvantage of this technique is the need to execute the model a certain number of times to obtain the probability distribution, which might be unfeasible depending on the application. This work represented a turning point that led to an increased research interest in DL-based architectures targeting probabilistic forecasting [21]. Various architectures have been employed to follow this approach in time series forecasting. As an example, DeepAR is an encoder–decoder LSTM-based architecture that provides probabilistic forecasting by predicting a likelihood model (e.g., Gaussian likelihood). It uses MCDO to sample from the obtained probability distribution. The previous assumption of the modeled probability distribution is a limitation of this architecture [24]. As a matter of fact, the Gaussian assumption when modeling glucose variations over time is not effective [25]. In this sense, DeepPIPE introduces the interval prediction. Unlike DeepAR, this model does not make any prior assumption of the output probability distribution [24]. Similarly to DeepPIPE, the model presented in [26] employs quantile prediction for distribution-agnostic probabilistic multi-horizon prediction. Finally, Generative Adversarial Networks (GANs) have also been employed in this regard, modeling arbitrary probability distributions using only the generative part of this network [27].

The *black-box* nature intrinsic to DL architectures limits their implementation in scenarios where knowing the reasoning behind an automatic decision is mandatory. This is especially critical in healthcare, and, specifically, in glucose forecasting, where AI-aided guidance to avoid critical life-threatening glycemic levels should be understandable and interpretable [28]. EXplainable AI (XAI) has recently emerged as a research area to overcome this issue [29]. Aside from the direct benefits obtained from understandable AI models, information enrichment provided by XAI is associated with an increased adherence to mobile Health (mHealth) tools, whose continuous usage usually implies improved health outcomes [20,30]. Focusing on interpretable time series forecasting, post hoc techniques, fuzzy logic, and attention mechanisms are the most widely used methods [31]. Transformers are based on attention mechanisms and have shown superior performance in the sequence-to-sequence (*seq-to-seq*) paradigm (i.e., an input sequence produces an output sequence) like Natural Language Processing (NLP) approaches [32], being the base of models such as ChatGPT [33]. Since personalized glucose prediction can be considered a *seq-to-seq* task, a transformer-based architecture has been employed in this work to tackle it by also providing model interpretability and uncertainty estimation.

Nonetheless, the study by Zeng et al. demonstrated that the use of transformers for time series forecasting does not always outperform simpler algorithms. Among other experiments, they developed a linear model that outperforms most of the transformers studied for time series forecasting, also proving that, when shuffling input data, performance was not harmed. This suggested that transformers did not learn temporal dependencies [34]. However, the Temporal Fusion Transformer (TFT) [35], which includes LSTMs for local temporal processing, was not assessed in the previous study. The TFT has demonstrated an ability to identify temporal patterns within a wide variety of datasets, outperforming classic autoregressive approaches, like ARIMA [36] or modern DL architectures, such as DeepAR [24], setting the state of the art in interpretable, probabilistic forecasting. In addition, TFT architecture allows us to process heterogeneous data, from the time series itself, numerical variables like the day of the month, or categorical variables like subject identifiers, among others [35]. The latter is especially relevant for this work, since it enables personalized glucose prediction.

Zhu et al. [37] previously used the TFT for glucose prediction using CGM data. Although they achieved positive results, some limitations were identified. Firstly, they did not evaluate how varying input features influenced model performance, interpretability, and uncertainty. In fact, they did not quantify model uncertainty in any way, nor the interpretability of the model. Additionally, this work did not include a subject identifier as an input feature, hindering a personalized approach in the prediction. Finally, the model was tested with datasets that did not include more than 14 days of CGM readings in any case, which does not include yearly–seasonality pattern changes.

All these limitations found in Zhu et al.’s work (namely, an exhaustive study regarding feature contributions in prediction performance, attention variations within the input sequence and quantitative uncertainty estimation) were tackled by our study. In addition, an incremental study (i.e., adding one variable at a time in the training stage of the TFT) has been assessed to analyze changes in prediction performance and model behavior, leveraging the in-built capabilities of this model. To the best of the authors’ knowledge, this is the first time that such an exhaustive study has been carried out using the TFT to target personalized glucose prediction. Thus, our work proposes the use of the TFT for personalized and interpretable probabilistic interstitial glucose level forecasting. This approach aims to provide the potential end-user (i.e., a person with T1D) with deeper insights into the AI-based predictions on their short-term glucose level estimation, as well as (a) how *certain* such an estimation is (including upper and lower probability distribution bounds) and (b) *why* the model has made such a prediction. With this goal, different experiments were carried out, varying the TFT input features using two datasets (including a reference dataset for glucose forecasting evaluation), analyzing how the model behaved in each case.

## 2. Materials and Methods

### 2.1. Datasets

#### 2.1.1. WARIFA Dataset

The WARIFA dataset, named after the European Project from which the data were collected [38], only contains CGM readings with their associated timestamps and an anonymized subject’s ID. Variables such as insulin administration, carbohydrate intake, or physical activity were not available for any of the subjects involved in this study. Data were collected at the *Complejo Hospitalario Universitario Insular-Materno Infantil de Las Palmas de Gran Canaria* from 41 subjects with T1D using a sensor from the Abbott FreeStyle Libre (Abbott Laboratories, Abbot Park, IL, USA) family. Participants were invited to participate and were given oral and written information. All participants signed a written informed consent form. The study was deemed exempt from assessment by the local ethics committee, since it did not fall within biomedical research regulations. An endocrinologist extracted the CGM data in raw CSV (Comma-Separated Values) format from the Abbott LibreView^TM^ (Abbott Laboratories, Abbot Park, IL, USA) website.

To be able to evaluate the models using a whole year of data, only the subjects that met the following inclusion criteria were considered, as performed in previous work [39]:

1. Wearing the same CGM sensor for at least one year.
2. Wearing a CGM sensor with a sampling period of 15 min.

#### 2.1.2. OhioT1DM Dataset

The OhioT1DM dataset [40] can be considered the reference for glucose forecast benchmarking. Therefore, it has been incorporated in this study for comparison with existing methods, and to evaluate how the TFT performs with two T1D-related datasets that include different variables. This is a curated dataset that comprises two data collection campaigns that took place in 2018 and 2020, containing eight weeks of data from 12 subjects with T1D with associated, anonymized IDs. The OhioT1DM dataset, provided upon request in XML (Extensible Markup Language) format, included subjects who wore an insulin pump, and contained 20 variables for each subject, comprising CGM readings every five minutes, physiological data, and self-reported data [40]. However, this study focused on the variables considered more relevant for personalized glucose prediction: *CGM readings*, *ID*, *heart rate*, *carbohydrate intake*, *basal insulin rate* (the rate at which insulin is infused into a subject), *bolus insulin* (instantly delivered insulin dose, used before meals and for corrections), and their *associated timestamps*. It is worth noting that, in the anonymization process, all dates associated with the CGM readings were shifted to a random time in the future but keeping the times of the day and the days of the week, making the use of such temporal data impossible as input features. The subjects included in the study wore Medtronic 530 G or 630 G insulin pumps and used Medtronic Enlite CGM sensors (Medtronic, Fridley, MN, USA). Physiological data that included heart rate, skin temperature, sleep, steps and acceleration were collected from a fitness band: Basis Peak (Basis Science Inc., San Francisco, CA, USA) in the 2018 cohort, and Empatica Embrace (Empatica Inc., Boston, MA, USA) in the 2020 cohort.

### 2.2. Data Preparation and Partition

#### 2.2.1. WARIFA Dataset

After filtering out the subjects that did not meet the inclusion criteria, there were 29 different CGM sequences (1 per subject) with their associated timestamps containing one year of glucose readings. These sequences were adapted to train the TFT following a *seq-to-seq* approach, namely an input sequence that outputs an estimated future sequence (in this case, a future sequence of prediction intervals). As performed in [39], all subjects’ sequences were swept into one-step intervals to generate the model input sequences, including 96 samples, and output sequences, including 4 samples, representing a prediction horizon (PH) of 60 min at a sampling period of 15 min. Although the nominal sampling period of all sensors was 15 min, the actual period between samples could vary slightly from this reference value. Thus, when the difference between two consecutive timestamps surpassed 29:59 min (2 × sampling periods minus one second), it was considered a reading interruption, and a sequence was not generated for such timestamps. The work in [39] reported histograms of the intervals between consecutive readings for all subjects, which justified that this threshold did not compromise temporal consistency while allowing the generation of a sufficient number of samples. In this work, an interruption implied that a new sequence was generated, and data imputation was not performed so models were trained only with full CGM patterns. Once all subjects’ input and output sequences were generated, they were concatenated in a subject-wise scheme to perform data partition. The optimal input and output sequence lengths of the models were heuristically obtained in [39]. In addition, the same study demonstrated that the definition of the interruption allowed a sufficient number of instances for this dataset, without allowing significant temporal inconsistencies. For the sake of fair comparison, this work replicated this preprocessing.

The generated sequences, which comprised data from a whole year, were divided into four trimesters. For all subjects, each trimester was randomly split into 60% for the training set, 20% for the validation set, and 20% for the test set, following a stratified approach ensuring that all seasons, days, and months were present on each partition (Figure S1). Performing this for all subjects and concatenating all sequences constituted the final training, validation, and test sets. Sequences that contained samples from two different trimesters were excluded from the subsets to avoid including the same datapoints in different sets of data (training, validation, or test). Although the number of samples that contributed to the final sets varies for each subject depending on the number of reading interruptions, this partition ensures a balanced representation of all seasons in all subsets for all subjects. This guarantees that the model is trained, validated, and tested with data from all seasons, likely capturing changes in glucose variation patterns associated with particular events that occur during the year.

#### 2.2.2. OhioT1DM Dataset

Unlike the WARIFA dataset, the OhioT1DM dataset provides data from heterogeneous sources. This implies that the timestamps between input variables (e.g., between a given CGM reading and a carbohydrate intake) rarely match. Heart rate values were discarded due to their unavailability in some of the subjects, so CGM timestamps were considered the reference to generate a common time grid to be able to feed the TFT with features sampled at slightly different time instants. The initial CGM timestamp was the first element of a common time grid, whereas the last one was the last element of the common time grid. The interval between consecutive timestamps in the common time grid was 5 min, since it was the sampling period of the CGM sensors included in this dataset.

From here, the closest match between the timestamps from the CGM and the rest of the input features was assigned to the common time grid (Figure S2). When the original CGM timestamp did not properly fill the common time grid (i.e., for two consecutive timestamps, the same closest match was found), it was considered an interruption. For the rest of the variables, when a value was not read, it was set to zero in the corresponding timestamp in the common time grid, except for the basal rate, whose value was kept until updated by the immediate next timestamp. This generated a long sequence from which the input and output sequences to train the TFT, following a *seq-to-seq* approach, were generated.

Regarding dataset partition, the train and test sets provided by the authors and commonly used in the literature were employed. The authors only specified training and test sets, so the training set was subdivided as training and validation in an 80-20 partition scheme, ending up with 74,121 (65%), 18,530 (16%), and 21,020 (19%) samples for the training, validation, and test subsets, respectively.

### 2.3. Temporal Fusion Transformer

The TFT is an encoder–decoder transformer-based model that was designed for multi-horizon probabilistic forecasting, providing model interpretability. Rather than single-point predictions, this model computes probabilistic predictions: it estimates prediction intervals by adopting quantile regression for each time step. In other words, it predicts the conditional quantiles of the target distribution [26,35] (i.e., in this case, a range within which all possible future glucose values could fall). This is essential to enrich the information provided to a subject to, for example, avoid a hypoglycemic episode. The TFT also enables the combination of heterogeneous data to perform predictions: observed past inputs (e.g., past CGM readings), exogenous time series that influence the future of the target variable (e.g., heart rate), and static covariates (e.g., subject ID or medical metadata, such as weight) [35].

TFT relies on attention mechanisms to enhance interpretability [35]. An attention function maps a *query* and a set of *key–value* pairs to an output [32]. Intuitively, the query can be seen as the input CGM sequence embedded with its additional covariates (subject ID, heart rate, carbohydrate intake, etc.), and the key–value pairs as the possible values that the output sequence can take, together with its likelihood. These key–value pairs, obtained after training, give an idea about what the model is focusing on to obtain the final prediction, enabling interpretability of the output. To accomplish this, TFT implements a multi-head interpretable attention mechanism that shares weights within attention heads, introducing additive aggregation within them. In addition to the aforementioned features of the TFT, its most important components are listed and briefly explained below [35]:

(1) **Gating mechanisms** to adapt network complexity for a given dataset by skipping unused components of the architecture (e.g., if a dataset does not contain static covariates, the corresponding encoder will not be present in the final implementation of the model). This provides flexibility to perform different experiments to analyze the impact of a given input feature on model performance without further changes in the model.
(2) **Variable selection networks** to select the most relevant input features at each time step.
(3) **Static covariate encoders** to condition temporal dynamics through the integration of the static covariates.
(4) **Temporal processing** to learn long-term (through multi-head attention layers) and short-term (through LSTM) temporal relationships.

### 2.4. Experiment Design

The design of the experiments presented in this work has taken into account the capabilities of the TFT to provide model interpretability and in-built variable importance estimation. Inspired by the ablation study performed in [35], the input variables to train the TFT were added incrementally in subsequent experiments to compare the prediction performance, input feature importance, and model uncertainty between the different cases. For both datasets, the TFT trained solely with CGM readings was considered the baseline. This incremental study is clinically meaningful since certain glucose level patterns might be associated with a specific time of the day (e.g., at a given hour a subject always exercises), day of the week (e.g., diet can vary slightly during the weekends), or the month (e.g., holiday period), having meaningful impacts on glycemic control [41,42]

Pursuing a personalized prediction, each subject was treated as an *entity* with its associated covariates and inputs, as the TFT has been proven to effectively distinguish between individual elements using this technique [35]. This was performed by treating the ID as a categorical static covariate. Thus, for both datasets, the second experiment consisted of adding the ID as an input variable, so the model associated the encoded value as a subject with her/his associated CGM reading.

Since the WARIFA dataset only includes CGM readings and their timestamps, and to maintain consistency between the experiments in both datasets, the temporal information was added immediately after the ID. This information was extracted from the timestamps. The hypothesis underlying the order of the addition of the temporal variables is that the greater the time granularity (i.e., hour of the day and day of the week), the more valuable the information provided to the model, although the month could be associated with changes in glucose patterns related to a specific season. Once the temporal information was included in the model, the T1D-related variables were incrementally added, though this was only possible using the OhioT1DM dataset. The order of such variables was based on the information provided to the model. Since the basal insulin rate was never zero, it was the first feature to be added. Furthermore, a slightly higher number of carbohydrate intake episodes was observed compared to the number of bolus insulin administrations. Subsequently, bolus insulin was the last added variable, just after the carbohydrate intake. Table 1 compares both datasets and summarizes the order in which the variables were incrementally included as input in the TFT. Additionally, it outlines which variables were available in each dataset, how those variables were included in the TFT, the sensor sampling period, the number of involved subjects, and the number of experiments carried out with each dataset. Given the noticeable differences between datasets regarding temporal granularity, differences in diabetes-related variables and number of included subjects, the evaluation and comparison of the obtained results could be valuable to gain insights into which variables are more helpful to obtain a precise glucose prediction with low uncertainty using the TFT model.

Table S1 shows the number of instances in each subset for the data partition for both datasets. Notice that the number of available training instances in the WARIFA dataset is around 3.5 times larger than its analog for the OhioT1DM dataset. This difference may limit model performance for the latter case, although more input features are available. Test instances were used to evaluate the models with unseen samples not used during the training phase.

### 2.5. Model Training

The experiments were carried out using the Pytorch Forecasting library 1.2 [43], which works on Python 3.9 [44] and Pytorch 2.5.1 [45]. All experiments were executed on an AMD Ryzen 5 3600 6-core processor (Advanced Micro Devices, Inc., Santa Clara, CA, USA) and an NVIDIA GeForce RTX 4070 Ti GPU (NVIDIA Corporation, Santa Clara, CA, USA). For consistency in the comparison with previous work using the WARIFA dataset, the input sequence length was set to 96 samples, corresponding to one day of CGM readings [39]. In the OhioT1DM dataset, this parameter was also intended to include one day of data (288 samples), but it significantly reduced the number of training instances. Hence, for consistency between experiments, this parameter was finally fixed to 96 samples, corresponding to eight hours of CGM readings. The selected PH was 60 min, as it is common in the literature and clinically relevant. This corresponds to 4 and 12 samples in the predicted sequence for the WARIFA (sampling period=15 min) and OhioT1DM datasets (sampling period=5 min), respectively. As performed in [35], the selected prediction interval percentiles for each time step were 10th, 50th, and 90th. Therefore, the predictive interval will ideally cover 80% of the most feasible future glucose values for each predicted time step, including the average tendency (corresponding to the 50th percentile). This is important in a context where extreme cases, namely hyper- and hypoglycemic episodes, should be predicted accurately to properly assist subjects’ decisions to prevent health-threatening events.

After data partition, all data subsets were normalized using Z-score and then shuffled to feed the TFT. The batch size was set to 512 using the WARIFA dataset, due to the large amounts of available instances, saving computational time and trying to avoid overfitting. In the experiments with the OhioT1DM dataset, the batch size was fixed to 128 to compensate for the lower number of available instances. Hyperparameter optimization was performed using Optuna [46]:

- Number of attention heads.
- Hidden size (common within all TFT DL layers).
- Hidden size to process continuous variables.
- Maximum gradient norm (i.e., the maximum value a gradient update can have).
- Learning rate.
- Drop-out rate.

The optimization process was performed using the Tree-structured Parzen Estimator (TPE) algorithm [47,48]. The TPE algorithm is a Bayesian optimization method that fits a Gaussian Mixture Model (GMM) to the set of parameters associated with the best case, and another GMM to the remaining parameter values, selecting the values that maximize the ratio between both GMMs. Since the search space of the TFT is large and continuous, 25 trials (i.e., different hyperparameter optimizations training the TFT from scratch) were run for each experiment to find the best hyperparameter combination. Each trial runs 100 epochs. This is a highly time-consuming task, so, considering the number of experiments run in this study, the median pruner [49] was employed to save computational time by discarding unpromising trials. The median pruner uses the median stopping rule: if the best intermediate result of the current trial is worse than the median of the previous trials at the same point, the trial is interrupted [49]. Additionally, the minimum number of trials before starting pruning was set to five. The search space for each hyperparameter was based on prior work [32,35], and is specified in Table S2. The TFT was trained with the Adam optimizer to minimize the quantile loss [26], based on Equation (1), which is summed across all outputs for all PHs (in this work, 10th, 50th, and 90th percentiles), where y is the true value, $\hat{y}$ stands for the predicted value, q represents a certain quantile, and the $(\cdot {)}_{+}$ operator computes the maximum between 0 and ‘·’. The code of these experiments is available in a public repository for reproducibility (available at https://github.com/antorguez95/TFT4GlucosePrediction, accessed on 13 June 2025).

(1) $$QL\;(y,\;\hat{y},\;q)=q(y-y{)}_{+}+(1-q)\;(\hat{y}-y{)}_{+},$$

### 2.6. Evaluation Metrics

#### 2.6.1. Deterministic Metrics

Classical regression metrics, namely, Root Mean Squared Error (RMSE), Mean Absolute Error (MAE), and Mean Absolute Percentage Error (MAPE) (Equation (S1), Equation (S2), and Equation (S3), respectively) were computed using the 50th predicted percentile (i.e., the median of the estimated predicted distribution) to enable comparisons with existing methods. These metrics were evaluated using only the last sample, corresponding to the largest PH (i.e., 60 min).

#### 2.6.2. ISO-Based Metrics

Although deterministic metrics provide valuable information about model performance, they are not specifically tailored to the diabetes-specific prediction problem. It has been demonstrated that better regression metrics do not always imply better prediction performance in the diabetes context [39]. Hence, two metrics based on the ISO 15197:2015 standard [50], which establishes the minimum requirements for glucose monitoring devices to be considered clinically safe, have been assessed in this work:

- First, 95% of the measured (in this context, predicted) glucose values must be within ±15 mg/dL for blood glucose concentrations below 100 mg/dL. For values equal to or greater than 100 mg/dL, the margin of error is fixed to ±15% of the reference value. The metric we call *ISOZone* represents the number of points that fall within this range.
- Second, 99% of the measured (in this context, predicted) glucose values should fall within zones A and B (considered clinically safe) of the Consensus Error Grid (CEG) for T1D [50]. The metric we call *ParkesAB* indicates the number of points that meet this requirement.

#### 2.6.3. Uncertainty Metrics

To estimate model uncertainty in the predicted distribution, the normalized quantile loss, described in Equation (2), was evaluated on the predicted percentiles (10th, 50th, and 90th), where $\tilde{\Omega }$ is the domain of the test samples, $y_{t}$ a test sample at time *t*, τ the prediction horizon, and *QL* the quantile loss described in Equation (1).

(2) $$q-Risk=\frac{2\sum_{y_{t}\in \tilde{\Omega }}\sum_{\;\tau =1}^{{\;\tau }_{max}}QL\left(y_{t},\;\hat{y}\left(q,\;t-\tau ,\;\tau \right),\;q\right)}{\sum_{y_{t}\in \tilde{\Omega }}\sum_{\;\tau =1}^{{\;\tau }_{max}}\left|y_{t}\right|}$$

This metric quantifies the accuracy of the quantile of the predictive distribution, enabling the approximation of the uncertainty in the prediction [26,35]. The lower its value, the less uncertainty in the predicted quantile.

#### 2.6.4. Interpretability Evaluation

By leveraging the in-built interpretable capabilities of the TFT [35], it is possible to estimate which input features and which time instants of the input sequence are more relevant to perform a probabilistic prediction. Attention and feature importance were compared between experiments to analyze how the model behaved when additional features were used during training. In addition, an instance-wise evaluation was conducted to qualitatively evaluate the uncertainty of random predictions, due to the large amount of available test samples.

## 3. Experimental Results and Discussion

### 3.1. Prediction Performance and Uncertainty Estimation

Table 2 and Table 3 show the deterministic (RMSE, MAE and MAPE), ISO-based (*ParkesAB* and *ISOZone*), and uncertainty metrics (*p10*, *p50*, and *p90*) obtained after evaluating the generated TFT models with the WARIFA and OhioT1DM test sets, respectively. Moreover, for each model, the number of parameters was extracted to evaluate how the size of the model varied when features were included as inputs.

For both datasets, a more accurate prediction of the average tendency (reflected by a decrease in the deterministic metrics) generally implied less uncertainty in such predictions (i.e., lower q-risks). This means that prediction accuracy improves as *epistemic uncertainty* decreases, which is desirable.

#### 3.1.1. Results of the WARIFA Dataset

Focusing on the results obtained with the WARIFA dataset (Table 2), the *subject ID*, included as an exogenous input feature to enable personalized prediction, significantly improved the prediction of the average tendency and decreased model uncertainty with respect to the baseline model (only trained with *CGM readings*). Similarly, the addition of the *hour* as input improved all metrics. However, the inclusion of the *day of the month* and the *day of the week* did not show any improvements, slightly worsening all the metrics in the latter case. Interestingly, the addition of the *month* as an input to the TFT showed the best prediction performance and the lowest prediction uncertainty within all experiments. This model decreased the baseline RMSE, MAE and MAPE by 32%, 37%, and 37%, respectively. This means that the TFT trained with *CGM readings*, the *subject ID*, and temporal data (that comprises *hour*, *day of the week*, *day of the month*, and the *month* of each reading) estimates the average tendency of the predicted distribution significantly better than the rest of the input feature combination. Equivalently, *p10*, *p50* and *p90* decreased by 24%, 29% and 27%, respectively. Hence, the predicted distribution of the best model is considerably more accurate than the baseline, providing predictions with a lower degree of uncertainty. This is crucial in the context of glucose prediction, where a wrong estimation of critical glucose levels (i.e., hyper- and hypoglycemic values) could mislead a subject into wrong actions that can end in health-threatening events. The fact that the inclusion of the *month* showed the best results suggests that introducing more seasonality information to the model helped the TFT to better learn the context to analyze CGM patterns depending on the day and the month of the reading. This is further discussed in Section 3.3.1.

Analyzing the diabetes-specific metrics, it is remarkable that, except for the baseline model, the *ParkesAB* always reached or surpassed 99%, fulfilling one of the ISO requirements. Conversely, the criteria associated with the *ISOZone* metric were not fulfilled in any case. Nonetheless, the model that included the *month* as an input feature presented the highest value (85.13%), being close to the 95% required by the ISO standard. Considering that only CGM readings and their timestamps have been used to train the TFT, this result suggests that this approach could meet both ISO requirements by including additional diabetes-related features like those present in the OhioT1DM dataset.

Finally, the addition of input features generally implied larger models. Nonetheless, the best model, which also includes the *month* as input, is slightly lighter than its two immediate predecessors within the experiments. This is mainly because, although more input variables were present, the number of attention heads obtained after hyperparameter optimization was lower (3 against 4 and 8). Considering that the TFT was trained using a 32-bit floating point, model sizes ranged from 15.76 MB to 19.49 MB. Specifically, the best model would require 19.12 MB of storage, which is not critical in the context of mHealth tools, usually executed on smartphones or in cloud services.

#### 3.1.2. Results of the OhioT1DM Dataset

The test results obtained using the OhioT1DM datasets showed a general prediction performance downgrade compared to the WARIFA dataset results (Table 3). This can be partially explained by the differences between the datasets. First of all, although the input sequence length is the same, sensors included in the OhioT1DM dataset have a sampling period three times lower (i.e., higher sampling rate) than the WARIFA dataset. Hence, OhioT1DM instances comprise 8 h of CGM data, compared to the 24 h of the WARIFA instances, providing more granular information, but less mid-term context in the input sequences. Additionally, the number of subjects included in the OhioT1DM dataset is less than half that of the WARIFA dataset (12 against 29 subjects). This, together with the fact that the monitoring period is also substantially shorter (i.e., the number of training instances per subject is drastically lower, as presented in Table S1), might potentially limit the ability of the TFT to recognize *entities* (i.e., subjects) enabled by the inclusion of the *ID* as an input variable. Finally, the length of the predicted output sequence is 12 samples, i.e., three times more than in the WARIFA dataset. In this sense, sampling at larger rates and predicting longer sequences inherently implies more noise, model uncertainty and error accumulation in sensor measurement and model predictions [51]. This is reflected in the q-risk computation, since it considers the whole output sequence regardless of its length. Hence, higher values in the q-risks are expected.

Table 3 shows that neither *ParkesAB* (>99%) nor *ISOZone* (>95%) ISO requirements were fulfilled. This means that the model trained with the OhioT1DM dataset is not close to being considered reliable in the glucose prediction context, since it could lead to wrong and dangerous actions regarding glycemic control. Furthermore, performance degradation after the inclusion of the *subject ID* as a model input suggests that, due to the low number of subjects included in this dataset, monitored only for two weeks, the TFT might not have properly recognized subjects as independent *entities*. Regarding temporal information, the inclusion of the *hour* as a numerical variable slightly improved the baseline (only using *CGM readings*) in terms of prediction performance of the average tendency and model uncertainty. Conversely, the addition of the *day of the week* downgraded the baseline. This, together with the results shown in Table 2 with the WARIFA dataset, suggests that the *day of the week* provides valuable context information to the model together with the *month*, but not on its own.

Analyzing the effects of the exogenous diabetes-specific variables, the inclusion of the *basal insulin rate*, a continuous variable that keeps a constant value until updated (normally after a few hours), also worsened baseline performance. On the contrary, the models resulting from the inclusion of both *carbohydrate intake* and *bolus insulin* significantly outperformed the rest of the models. Although their vectors are sparse (i.e., most of their values are zero), the major influence of these variables on glucose dynamics, which is clinically proven [52], was interpreted by the model, as further demonstrated in Section 3.3.2. Except for the p10 metric, which is barely lower than in the model where only *CGM readings*, *ID*, and *hour* are present (0.095 vs. 0.096), the model that includes *bolus insulin* notably outperformed the rest, achieving a *ParkesAB* that almost reached the ISO-established minimum (97.2% against 99.0%). Nonetheless, this improvement is mainly limited by the short monitoring period and the low number of subjects involved in the OhioT1DM dataset.

In terms of model uncertainty, described by the q-risks, the best model nearly tripled their analog in the WARIFA-trained model. Considering that the number of predicted samples is also triple, the best models for both datasets can be considered similar in terms of model uncertainty. In practice, this means that, although the average tendency (*p50*) is less accurate (i.e., deterministic metrics are higher), the predicted distribution of training with the two datasets is similarly reliable, both presenting low uncertainty. However, given the fact that ISO-based metrics are far from the standard minimum requirements, the models trained with the OhioT1DM dataset cannot be considered safe in the diabetes context.

Finally, more input features did not imply larger models, and more parameters did not ensure better performance, especially using a small dataset like the OhioT1DM dataset. The resulting model sizes were equivalent to those obtained with the models trained with the WARIFA dataset, with the best one requiring 18.22 MB of memory storage.

### 3.2. Analysis of the Model Interpretability

**Figure 1:**
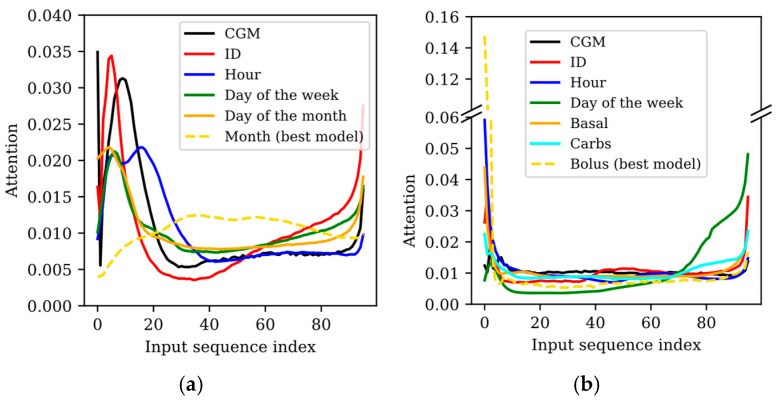
Attention over the input sequence (N=96) for all experiments carried out with the (**a**) WARIFA and (**b**) OhioT1DM datasets. Sample 0 corresponds to the first sample of the input sequence (i.e., 24 and 8 h before the moment of the prediction for the WARIFA and OhioT1DM datasets, respectively). Sample 95 represents the last data sample before the prediction (i.e., 15 and 5 min before the moment of the prediction for the WARIFA and T1DM Ohio datasets, respectively). Attention corresponding to the best case is highlighted with a dashed line. (Basal: basal insulin rate; Carbs: carbohydrate intake; Bolus: insulin bolus.).

The attention matrices provided by the TFT indicate where the model focused on within the whole input sequence to perform a prediction. Although one attention matrix is generated per predicted sample (four when training the TFT with the WARIFA dataset and twelve when training with the OhioT1DM dataset), the attention did not significantly vary between them. Hence, for simplicity, the attention corresponding to the first predicted sample has been analyzed for each experiment. Figure 1 shows the attention within the input sequence for all experiments (*N* = 96 for both datasets), highlighting the combination of features previously described that showed the best prediction performance for each dataset with a dashed line.

#### 3.2.1. Model Interpretability with the WARIFA Dataset

Figure 1a draws the TFT’s attention to the model input sequence after training the TFT with different sets of input features using the WARIFA dataset. Except for the best case (which includes the *month* as input), the model mainly focuses on the (approximately) first 20 and last 10 samples, as shown by the peaks at the beginning and end of the attention graph. When including only *CGM readings* and *CGM readings* with *ID*, this phenomenon is more drastic. The first samples are around 6 times more relevant for the final prediction than most of the remaining input sequence. This difference is alleviated when introducing the *day of the week* and the *day of the month*. However, such differences are also noticeable, since the attention at the beginning of the sequence nearly doubles its analog in the whole central part. In general, attention significantly increases in the last 10 samples, except for the best case. Intuitively, this might be expected, assuming that the most recent values are the most relevant for extracting information for the prediction. The fact that two thirds of the sequence is significantly less relevant for the prediction than the beginning and the end of the sequence suggests that the model is leveraging the full sequence containing 24 h of CGM readings in a significantly imbalanced way. The fact that the larger these differences are in attention, the worse the prediction performance is in terms of prediction accuracy and uncertainty (see Table 2) suggests that such an imbalance could be related to performance loss.

This is more evident by analyzing the best case, when the *month* is included as an input variable of the TFT. Prediction performance significantly outperforms the rest of the cases (especially the first ones, where the attention varies more drastically), and the attention curve is the smoothest one. The highest attention is approximately triple the lowest one, but there are not drastic changes between the attention of neighboring input samples like in the rest of the cases. Additionally, the samples with less associated attention are the ones at the beginning of the input sequence, but their importance is not negligible compared to the rest. Unlike the rest of the experiments, the TFT considers the whole central part of the sequence relevant. However, attention slightly drops at the end, even though an increase with respect to the rest of the sequence could be expected. Hence, the best prediction performance is directly related to the most balanced attention within the whole input sequence.

The results shown in Table 2 and the attention over the input sequence illustrated in Figure 1a suggest that including the month as an input feature helped the TFT to learn a broader context regarding CGM prediction, differentiating patterns that belong to a specific season of the year.

This feature of the TFT has a potential clinical utility: physicians could create a personalized plan to avoid critical glycemic episodes based on such attention plots. Nonetheless, current attention computation is global, not personalized. In addition, this attention corresponds to the input of the sequence regardless of its timestamps. Thus, further research is needed to study how this can be applied to clinical practice in an effective and secure way. Nonetheless, despite the potential of interpretability features, these must be clinically validated to ensure their safe use as a guide for endocrinologists or people with T1D. Additionally, over-reliance of the obtained results should be avoided with safeguards derived from clinical or pilot studies.

#### 3.2.2. Model Interpretability with the OhioT1DM Dataset

Figure 1b illustrates the attention over the input sequence after training the TFT with the different sets of input features in the OhioT1DM dataset. Although similar attention patterns are observed, the differences between datasets are translated into certain differences in the model attention. With the OhioT1DM dataset, an initial peak (more attention paid to the beginning of the sequence) is observed in all experiments. This peak is lower when the model is solely trained with *CGM readings* and with the introduction of the *day of the week* (i.e., two of the worst models). Specifically, the latter model, which presents the poorest prediction performance, is the one that focused the most on the last samples. This suggests that focusing mainly on the most recent data does not ensure good performance.

The aforementioned initial peak is especially exaggerated in the best model (i.e., the one that introduces the *bolus insulin*), where the first sample presents a value around 10 times higher than most of the middle part of the sequence. This shows that, although *insulin bolus* as input improved model performance, the context that comprises the full sequence has not been properly learned, as evidenced by metrics reported in Table 3. This might be related to the fact that the models are fed with only 8 h of data against the 24 h sequence of the WARIFA dataset. Apart from this abrupt difference, the attention of the best model presents the smoothest curve, showing a gradual increase at its end (i.e., more recent values), as could be expected. Furthermore, even though attention curves of all generated models increase at the end of the sequence, this change is especially abrupt in some of the worst-performing models (when including *ID* and the *day of the week*). On the contrary, the attention of the best three models (when *hour*, *carbohydrate intake* and *insulin bolus* are introduced) presents a similar pattern: a peak at the beginning of the sequence, balanced attention in its middle part, and increased attention at its end. The differences in the attention values in the middle and end of the attention vector are mainly determined by the initial peak (i.e., the attention associated with the first samples). Namely, larger peaks “leave” less attention for the rest of the sequence. The observed attention pattern suggests that the models present limitations in leveraging the information embedded in the whole input sequence. In these experiments, a smooth balanced attention pattern, such as the one depicted in Figure 1a for the best model of the WARIFA dataset, was not seen. This suggests that more balanced attention within the input sequence might be associated with better prediction performance.

### 3.3. Analysis of the Importance of the Features in the Model

**Figure 2:**
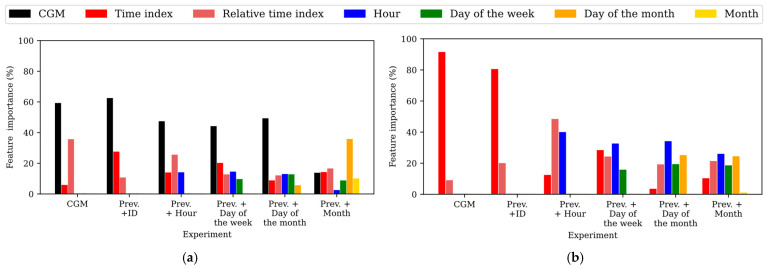
Feature importances expressed as percentages for all experiments involving training the TFT with the WARIFA dataset. Experiments are sorted from left to right in order of execution. (**a**) Feature importance in the TFT encoder. (**b**) Feature importance in the TFT decoder. *Prev.*: variables included in the immediately preceding experiment.

**Figure 3:**
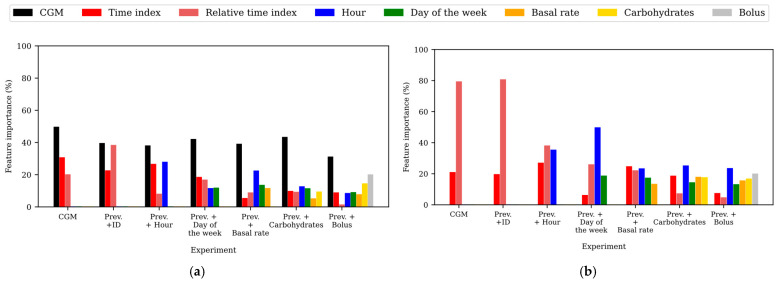
Feature importance expressed as percentages for all experiments involving training the TFT with the OhioT1DM dataset. Experiments are sorted from left to right in order of execution. (**a**) Feature importance in the TFT encoder. (**b**) Feature importance in the TFT decoder. *Prev.*: variables included in the immediately preceding experiment.

To gain insights into the relevance of the input features in the predictions, the feature importance within the different experiments training the TFT was assessed for the WARIFA and OhioT1DM datasets, as shown in Figure 2 and Figure 3, respectively. Notice that feature importance may vary from the TFT encoder, which learns the context and data patterns, and the TFT decoder, which generates the output sequence after having learned from the input data. As with the attention, feature importance barely varies within PHs, so the feature importances corresponding to the first predicted sample have been analyzed, being representative of the whole model. Additionally, *ID* does not appear in this analysis, since it is the only static covariate included in this study and its relevance cannot be compared with time-varying features. In this comparison, the *time index* refers to the position of a given sample within the input sequence with respect to the moment of the prediction, whereas the *relative time index* refers to the time index of a given sample with respect to the rest of the samples present in the input sequence. Furthermore, the variable *CGM reading* is not present in the TFT decoder, since it is the target variable (i.e., the prediction itself).

#### 3.3.1. Feature Importance in the WARIFA Dataset

Figure 2 shows the feature importance after training the TFT for both the *encoder* (Figure 2a) and the *decoder* (Figure 2b) using the WARIFA dataset. It can be observed that the *CGM reading* is always the most important feature in the TFT encoder except for the case of the best model, where the *month* is introduced as an input feature. This was expected, since *CGM reading* is the target variable. The importance of the *time index* and the *relative time index* decreases in both the encoder and decoder with the inclusion of more input features, yet their importance is generally never negligible.

Moreover, it can be observed that the importance of the most granular temporal information (i.e., the *hour*) is higher when generating the output sequence (decoding) than when encoding the information. This suggests that the TFT focuses on longer-term information to encode the input data, and on shorter-term information to perform a prediction. This is especially evident in the best case: the inclusion of the *month*, a variable that provides information about long-term seasonality. Although its importance is negligible in the decoder, its introduction produces an increase in the importance of the *day of the month* (which is the most important feature in the encoder), suggesting that it helps the model to learn long-term context.

Conversely, the *hour* becomes the most important feature in the decoder, its importance in the encoder being the lowest among all variables. This behavior supports the hypothesis that the encoder focuses on the longer-term patterns and the decoder on the shorter ones. In this line, the importance of the *day of the month* is higher in the encoder than in the decoder, in the opposite way to the *day of the week*. Additionally, *CGM reading* and time indices have similar importance in the encoder and decoder, while the *relative time index* is slightly more important in the decoder. This phenomenon, together with the prediction performance shown in Table 2, highlights the importance of introducing long-term information to offer more accurate interstitial glucose prediction using the TFT.

#### 3.3.2. Feature Importance in the OhioT1DM Dataset

Figure 3 illustrates the obtained feature importance during training the TFT for both the encoder (Figure 3a) and the decoder (Figure 3b) using the OhioT1DM dataset. As observed in the WARIFA experiments (except for the best case), the *CGM reading* is the most important variable in the encoder for all experiments. Additionally, the *time index* and the *relative time index* lost importance in favor of the successively added variables in both the encoder and decoder. This was expected, since the *hour* and the *day of the week* provide more accurate temporal information. In fact, the importance of the *relative time index* is negligible in the best case (the model that includes the *bolus insulin* as input).

It is noticeable how the *basal insulin rate* and the *carbohydrate intake* do not have a meaningful impact on the encoder by themselves, but their importance, especially that of the *carbohydrate intake* variable, is increased after the inclusion of the *bolus insulin*, which becomes the second most important feature in the encoder. Considering the results shown in Table 3, this suggests that the prediction improvements are associated with the information provided by carbohydrates and bolus variables that enable the TFT to better learn a diabetes-specific context like the glucose dynamics.

Analyzing the feature importance in the decoder, the *basal insulin rate* and the *carbohydrate intake* inclusion (before adding the *bolus insulin*) had a high impact on the decoder. However, only the latter implied a prediction performance improvement (Table 3). In this line, the *bolus insulin* was again the second most important feature in the best model, after the *hour*. Hence, the TFT focused significantly on the *carbohydrate intake* and *bolus insulin* to learn the data dependencies and patterns, also important in the decoder to generate the output sequence.

Furthermore, as with the WARIFA dataset, the short-term information, namely the *hour*, gained importance in the decoder. This supports the hypothesis that short-term information is more relevant in the decoder and long-term information (not present in this dataset) in the encoder.

### 3.4. Uncertainty Qualitative Analysis

**Figure 4:**
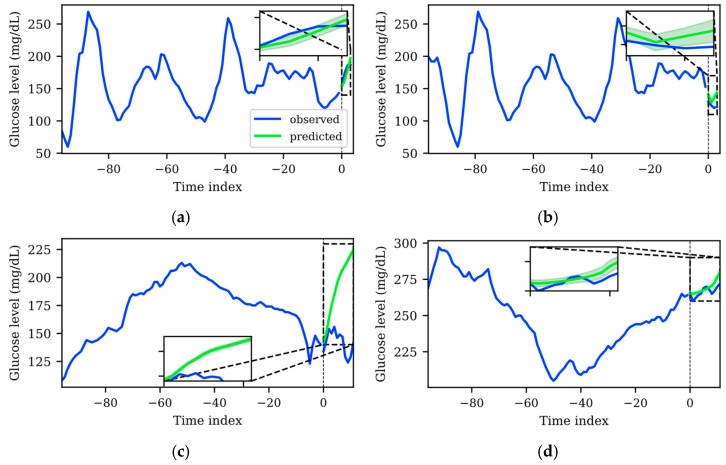
Representative examples of predictions with low uncertainty and high uncertainty for WARIFA (**a**), (**b**) and OhioT1DM datasets (**c**), (**d**), respectively. Predictions were generated with the best TFT models for each dataset. The blue solid line represents the input sequence and the output sequence used as the ground truth. The solid green line represents the average tendency of the prediction distribution (*p50*), whereas the shaded green area comprises the area between the upper and lower predicted quantiles (*p90*, and *p10*, respectively), representing *uncertainty.* The dashed black line in index 0 represents the moment of the prediction. Predicted sequences are zoomed for illustrative purposes.

One of the main objectives of this work is to train a TFT that provides accurate predictions with uncertainty estimation that will potentially help people with diabetes to make decisions based on the AI-based information. Hence, apart from the global analysis detailed in previous sections, a qualitative analysis of the prediction errors and their associated uncertainty was carried out. For this, instance-wise predictions were generated with the best model for each dataset, using their corresponding test sets, which were not used for the training and validation of the TFT model. Figure 4 shows two representative examples of the main findings of this analysis for both datasets, where the blue solid line represents the input sequence and the output sequence used as the ground truth, the green solid line represents the average tendency (*p50* of the predicted distribution), and the green shaded area represents the uncertainty of the TFT prediction (the upper (*p90*) and lower (*p10*) boundaries of the predicted distribution).

It is desirable that predictions whose average tendency is far from the ground truth are accompanied by higher uncertainty. This would mean that the model somehow recognizes less predictable patterns and assigns them larger uncertainty. This is in line with the fact that longer PHs and more drastic glycemic changes (i.e., larger prediction errors) imply more uncertainty. In the application scenario, this means that a person with T1D will be more cautious to make a decision based on the model output when a large, shaded area is shown.

Although there were specific cases that did not align with the abovementioned prediction error and uncertainty quantification compromise, such cases were significantly less frequent with the TFT model trained with the WARIFA dataset. This observation is consistent with the results shown in Table 2 and Table 3, which evidence that the TFT model generated using the WARIFA dataset provides more accurate predictions, also being more reliable in a diabetes-specific context. Figure 4a shows that the average tendency of the prediction distribution matches the ground truth. This prediction has a narrow shaded area, meaning a tight predicted distribution that implies low uncertainty. Equivalently, Figure 4b depicts a less accurate prediction, together with a broader prediction, which means more uncertainty in the prediction. Both cases demonstrate an appropriate behavior for the TFT model.

Conversely, the TFT model trained with the OhioT1DM dataset showed a significant number of patterns that were not consistent between prediction accuracy and uncertainty estimation. A representative case, especially relevant in the diabetes context, is illustrated in Figure 4c. A pronounced error between the prediction *p50* and the ground truth can be observed. However, the *p10* and *p90* bounds are very close, forming a narrow predicted distribution that suggests negligible uncertainty, which does not represent the real phenomenon. Additionally, this error occurs in a critical glycemic range. The prediction suggests, with a low degree of uncertainty, that a hyperglycemic event will arise within the next hour (glucose level >180 mg/dL). This estimation will lead to insulin administration to avoid this episode, potentially leading to dangerous severe hypoglycemia (glucose level<70 mg/dL). This example highlights the importance of correctly estimating uncertainty, so people with diabetes are aware of which estimations are more or less reliable. Figure 4d depicts the opposite case, where a predicted sequence that fairly resembles the ground truth shows higher uncertainty than the previously analyzed pattern. This shows that the model, in these specific cases (although this behavior was observed regularly in the OhioT1DM dataset), is not able to detect which patterns are less or more reliably predictable, being unsuitable for implementation in a real scenario.

### 3.5. Comparison with the State of the Art

To position this work within the state of the art in probabilistic glucose forecasting, a comparison with relevant studies was performed. Unfortunately, only one study has been conducted using the WARIFA dataset, based on the traditional deterministic approach using a Stacked-LSTM [39], so comparison against methods that use this dataset following a probabilistic approach was not possible. Regarding the OhioT1DM dataset, only studies contextualized within the scope of probabilistic 60 min prediction were included. For a fair comparison, studies that did not follow the data partition specified in the original paper [40] were filtered out. Table 4 shows a comparison of the results, including RMSE as the deterministic metric, the diabetes-specific figures used in this study, and the indication whether personalization in the prediction, interpretability analysis, and uncertainty quantification were tackled.

The Stacked-LSTM implemented in [39], where the WARIFA dataset was used, followed a deterministic approach and thus did not provide interpretability insights and uncertainty quantification. Since this work followed a *do-it-yourself* approach, one model was generated per included subject, providing personalization, whereas the proposed TFT achieved that using a single model. The RMSE metric was reduced by ~48% with respect to the previous work, whereas the *ParkesAB* and *ISOZone* metrics were improved by ~2% and 51%, respectively. It is worth noting that although the *ParkesAB* increase was slight, it allowed the TFT model to fulfill the ISO criteria associated with this metric, unlike the Stacked-LSTM. Furthermore, the RMSE and *ISOZone* metric (which represents the most restrictive ISO criteria) obtained a high improvement with respect to the previous work, evidencing the potential use of this technique for interstitial glucose forecasting based only on CGM data.

Regarding the published studies based on the OhioT1DM dataset, the TFT used in this study was previously employed by Zhu et al. [37]. However, the *subject ID* was not included to personalize the prediction, and the uncertainty quantification of such predictions was not assessed. Furthermore, although feature importance was evaluated, it was only performed for the TFT encoder, and attention within the input sequence was not analyzed, limiting model interpretability. Additionally, the reported RMSE included the full sequence, instead of evaluating only the 12th sample (that corresponds to the 60-min PH). Thus, for comparison purposes, the RMSE of the best TFT was computed in the same way, obtaining a value of 27.02 against the 32.3 (16% less) reported by Zhu et al., demonstrating that our proposed TFT model achieved better performance. Furthermore, diabetes-specific metrics were not evaluated in [37]. Similarly, the GluNet developed by Li et al. [13], which used a CNN to predict the average tendency and the lower and upper bounds of the prediction, achieved a global 60 min RMSE of 31.28 using the full sequence for the RMSE computation, also proving the superior performance of our TFT, which showed an RMSE that was 13% lower. Additionally, no diabetes-specific metrics were reported, and personalization, interpretability, and uncertainty quantification were not present in the reported analysis.

Hence, based on this comparison, it can be stated that the proposed method not only provides prediction performance comparable with the state-of-the-art models for probabilistic glucose prediction models. It also provides a more clinical and diabetes-oriented perspective that provides objective uncertainty quantification and model interpretability. These promising results and the interpretable nature of this approach show that it has greater potential than the rest of comparable studies to be implemented in, for example, an mHealth tool. Nonetheless, clinical validation is required to move towards that ultimate goal.

## 4. Current Limitations and Future Work

### 4.1. Dataset Harmonization for the Development of AI-Based Glucose Forecasting Models

This study has demonstrated that when using the TFT, a state-of-the-art model for interpretable and probabilistic prediction [35], a significant difference in performance was observed when modeling the same phenomenon using two different datasets. These discrepancies are mainly explained by the differences between datasets illustrated in Table 1: the number of included subjects, their monitoring time, the features included, and the sampling rate (sensor-dependent). Although the incremental study proposed in this work provides insight into the importance of a given set of variables in glucose level forecasting, this approach does not quantify how important each variable is by itself. These experiments show, for example, that the addition of the *month* helped the TFT to better understand the context together with the *day of the month*. However, due to the duration of the TFT’s fine tuning with our hardware setup (5 days on average) and the amount of experiments required for such analysis (63 and 31 experiments with the OhioT1DM and WARIFA datasets, respectively), it was not feasible to perform a sensitivity analysis that could help us understand how important each variable is individually (experiments would take around 470 days). Further experiments optimizing the hardware setup could drastically decrease training times to enable this type of extended and detailed analysis.

The OhioT1DM dataset [40] has unequivocally enabled significant improvements in the glucose forecasting research field during the last few years, especially in AI-based approaches. However, some of the main findings of this work, like the key role that the variable *month* plays in encoding CGM-related features, highlight the need for increasing the monitoring period while collecting CGM data to build robust predictive models. Indeed, the performance gap between models, as well as the inconsistency between model behavior when training with both datasets (e.g., key features in one dataset are not present in the other; see Figure 2 and Figure 3), evidences the need for standardized procedures for diabetes dataset collection. Dataset harmonization would reduce the burden that heterogeneous data preparation pipelines and discrepancies between features entail, allowing for consistency in experiments and model comparison. It would increase the applicability of an approach like the one presented in this study to broader populations (race, age, type of sensors, etc.). Currently, this is not feasible due to the drastic dataset heterogeneity. Thus, dataset harmonization would ultimately lead to a common framework to design more reliable AI-based diabetes management tools, applicable to broader populations [53].

### 4.2. Open Questions in AI-Based Glucose Forecasting Input Data

As previously introduced, the comparison between the results obtained using both datasets raises various questions regarding the input data requirements to effectively provide an AI-based robust glucose forecasting algorithm: (a) What is the optimal input window length for model generalization? (b) What is the minimum number of subjects for reliable predictions? (c) Is there any trade-off between sampling rate and model accuracy?

Focusing on the last point, the TFT model based on the OhioT1DM dataset is fed with one third of temporal information with respect to the WARIFA dataset (8 h against 24 h), and it outputs a sequence three times longer (12 against 4 samples). Predicting the 4th sample will have, in principle, less associated error and uncertainty than the 12th predicted sample. Intuitively, this scenario will lead to poorer performance. In [51], the authors analyzed the impact of the sensor sampling rate in an AI-based fall detection classification task, concluding that better classification results were achieved by applying data down-sampling (which is equivalent to decreasing the sampling rate). Unfortunately, no similar studies exist for the glucose forecasting task. Thus, a down-sampling of the OhioT1DM dataset would be useful to gain insights into the impact of the sampling period, and its associated levels of noise, on glucose prediction performance using the TFT.

Apart from adjustments such as a finer tuning of the TFT to enhance the training process and achieve better prediction performance, the inclusion of *minutes* as an input feature, or exploring different values of *N*, there is room for further analysis to enhance glucose prediction. Firstly, the TFT allows missing data treatment [35]. On the one hand, this would lead to the inclusion of a larger number of instances for training and would generate a more suitable model for a real-world scenario, where reading interruptions often occur. On the other hand, how this will impact prediction performance is something to be studied. Then, alternatives to the common time grid proposed in this work could be assessed. Moreover, the sensor model could be included as a categorical input variable to train the model, as well as the subject’s identifier. Sensor measurement robustness directly impacts the quality of glucose monitoring and the person’s ability to effectively manage the disease [54]. Hence, based on how introducing the subject identifier improved model performance, the model might identify the *noise* associated with each sensor (whose design can impact the final model performance) by stratifying underlying noise similarly to a specific sensor model. Related to this, variables that explain sensor degradation due to aging (i.e., date when a subject started wearing a sensor), calibration process (as a categorical variable), or other electronics-related aspects that have a direct impact on CGM reliability could also help the model as inputs to enhance prediction performance. Additionally, the inclusion of clinical variables or glycemic indices [55] (i.e., indices that numerically describe parameters such as glycemic variability) could help the model to predict changes in patterns associated with a subject with certain characteristics (e.g., a slow insulin pharmacodynamic [56]). Finally, the inclusion of the variable *sex* might allow the model to better distinguish glucose patterns associated with, for example, the menstrual cycle, which is a sex-dependent event [57].

Finally, a step further on this research would be a personalized assessment of the global TFT (i.e., by analyzing metrics and patterns subject-wise, stratifying by the *ID* variable), as performed in [39] for fully personalized models. This could enable personalized therapies to enhance glycemic control, also allowing people with T1D to gain insights into their glucose patterns, helping them manage their condition more efficiently. This enriched information, combined with the use of XAI methods, could potentially enhance adherence to the tool and, in the end, improve their health outcomes [20,30,58].

### 4.3. Assessing the Feasibility of the TFT for T1D Monitoring Applications

The TFT model sizes reported in Table 2 are not critical in the context of mHealth tools. However, hardware optimizations might be necessary when implementing it onto a low-end embedded system for in situ and portable data processing. After minor performance improvements with respect to those reported in this work, it would be feasible to implement the TFT model trained and validated with the WARIFA dataset in an mHealth application or in a closed-loop system for automated insulin infusion. This would have a direct and positive impact from different perspectives, compared to the implementation of a model trained with the OhioT1DM dataset, where CGM data were sampled every 5 min:

- Decreasing the sampling rate implies energy savings, which would prolong the lifespan of the CGM sensor batteries. This is related to fewer sensor replacements and, subsequently, fewer interruptions in the glucose level monitoring.
- Data generation would be three times lower for the same timeframe, so data storage (and its associated energetic and economic costs [59]) would be drastically decreased. Related to this, given the same input temporal window, generated models would require less memory and fewer computational resources to execute them.
- Achieving accurate predictions using only the CGM data would avoid the need for harmonizing heterogeneous timestamps and would also decrease the noise and reading interruptions associated with an increased number of sensor measurements.

Thus, if the abovementioned modifications in the TFT led to the achievement of an *ISOZone* metric of 95% (i.e., clinically secure actions can be taken based on the provided prediction), the proposed TFT-based prediction system might be tested to assess its clinical utility, ensuring its safety in the diabetes-specific context. Additionally, an in-depth analysis targeting the computational (i.e., economic) cost of model training and inference is necessary to evaluate the feasibility of implementing this model at a large scale.

### 4.4. Diabetes-Specific Loss Function Development

The implementation of a context-specific loss function to enhance model prediction is a commonly followed approach [60], also employed for glucose prediction [25,61], including a loss function based on the ISO 15197:2015 standard [39]. However, most of these adapted functions have been designed for deterministic prediction. To date, there is no ISO-adapted loss function for probabilistic prediction. This might be related to the fact that probabilistic loss functions tend to be more prone to mathematical instability than the deterministic ones [62]. Thus, the use of an ISO-adapted quantile loss function could improve TFT prediction performance, especially in terms of diabetes-specific metrics. Taking a step further, although the ISO 15197:2015 is a fair approximation to tackle this task, recent advancements in this field point to the need for a standard to specifically assess short-term glucose prediction [50]. By adopting such a standard, AI developers and researchers will have a unified framework for benchmarking models, being able to evaluate if a model is feasible to be implemented as an mHealth tool in a real-world scenario. This standard could estimate the reliability of the prediction by analyzing the errors between the estimation and the reference (that should be defined), considering the glucose concentration ranges, as performed in the ISO 15197:2015.

## 5. Conclusions

In this work, the TFT, a state-of-the-art model for probabilistic prediction with uncertainty estimation, has been trained with two diabetes datasets: the WARIFA dataset, and the OhioT1DM dataset. To the best of the authors’ knowledge, this is the first study that exhaustively analyzes attention and variable importance, and quantifies uncertainty estimation in glucose forecasting based on CGM data using the TFT with two different datasets. The conducted experiments consisted of incrementally adding, as model inputs, the features available on each dataset, evaluating how the model behavior and prediction performance changed by leveraging the in-built interpretable capabilities of the TFT.

The results obtained using the WARIFA dataset were significantly better than those obtained after training the TFT with the OhioT1DM dataset. The best model achieved state-of-the-art RMSE at a 60 min PH, a *ParkesAB* that surpassed the ISO 15197:2015 minimum requirement, and reached 85% in the *ISOZone* metric (whose minimum is 95% to meet the ISO criteria), being, to the best of the authors’ knowledge, the highest value reported to date.

Comparing the best models after training the TFT with both datasets, it is observed that the key features to enhance the prediction performance on each case (the *month* in the WARIFA dataset, and the *bolus insulin* and *carbohydrate intake* in the OhioT1DM dataset) were not present in their analogs. This observation evidences the need to find ways to harmonize T1D datasets. This would lead to more efficient training, making it possible to leverage models such as TFT, and moving towards accurate, interpretable and clinically safe glucose predictors.

Finally, the methodology employed in this work followed an XAI-based approach that relies on interpretability and model uncertainty estimation, providing insights into *how* AI-based diabetes management systems work and *why* they provide accurate results. This implies that the proposed TFT based on the WARIFA database could be potentially implemented as an mHealth tool, providing clinically safe guidance to people with T1D. In addition, the proposed methodology can be applied to similar tasks in the healthcare field that are based on wearable monitoring, such as cardiovascular event tracking [63].

## Figures and Tables

**Table 1:** Comparison between WARIFA and OhioT1DM datasets, considering only the variables selected to train and evaluate the TFT, following the order in which they were added, indicating also how such variables were introduced in the TFT. The monitoring period, the CGM sensor sampling period, the number of subjects included (*n*), and the number of experiments carried out with each dataset are also indicated. A tick symbol indicates that the variable was collected. A cross symbol indicates that a variable was not available. The *Variable Type* column indicates how a specific variable was introduced to the TFT.

Included Variable	OhioT1DM	WARIFA	Variable Type
CGM	✓	✓	Time-varying real (input and target)
ID	✓	✓	Static categorical
Hour	✓	✓	Time-varying real
Day of the week	✓	✓	Time-varying categorical
Day of the month	✕	✓	Time-varying real
Month	✕	✓	Time-varying categorical
Insulin basal rate	✓	✕	Time-varying real
Carbohydrate intake	✓	✕	Time-varying real
Bolus insulin	✓	✕	Time-varying real
Monitoring period	2 weeks	1 year	
CGM sensor sampling period	5 min	15 min	
*n*	12	29	
Total number of experiments:	7	6	

**Table 2:** Deterministic metrics (PH = 60 min) and uncertainty metrics (i.e., q-risks) of all the experiments carried out with the WARIFA dataset. q-risks are dimensionless. Results in bold indicate the set of input features with the best performance for deterministic and uncertainty metrics.

Input Features	Deterministic Metrics			ISO-Based Metrics		Uncertainty Metrics			TFT Parameters
	RMSE (mg/dL)	MAE (mg/dL)	MAPE (%)	*ParkesAB* (%)	*ISOZone* (%)	*p10*	*p50*	*p90*	
CGM reading	29.26	20.93	13.77	98.71	69.64	0.042	0.084	0.048	4,132,609
Prev. + ID	26.59	18.76	12.40	99.00	73.85	0.039	0.078	0.043	4,465,497
Prev. + Hour	23.24	16.06	10.64	99.30	79.30	0.036	0.069	0.037	4,907,208
Prev. + Day of the week	23.32	15.88	10.47	99.32	79.72	0.036	0.069	0.040	5,076,316
Prev. + Day of the month	24.22	16.85	11.17	99.18	77.63	0.036	0.072	0.039	5,108,758
**Prev. + Month**	**19.78**	**13.09**	**8.62**	**99.54**	**85.13**	**0.032**	**0.060**	**0.035**	**5,013,680**

**Prev**.: variables included in the preceding experiment.

**Table 3:** Deterministic metrics (PH = 60 min) and uncertainty metrics (i.e., q-risks) of all the experiments carried out with the OhioT1DM dataset. q-risks are dimensionless. Results in bold indicate the set of input features with the best performance for deterministic and uncertainty metrics.

Input Features	Deterministic Metrics			ISO-Based Metrics		Uncertainty Metrics			TFT Parameters
	RMSE (mg/dL)	MAE (mg/dL)	MAPE (%)	*ParkesAB* (%)	*ISOZone* (%)	p10	p50	p90	
CGM reading	43.24	31.92	22.02	96.21	50.23	0.098	0.119	0.101	4,840,380
Prev. + ID	44.33	32.85	22.93	95.87	49.09	0.097	0.123	0.103	4,694,258
Prev. + Hour	42.80	31.19	21.62	96.00	51.76	**0.095**	0.118	0.100	5,271,167
Prev. + Day of the week	44.58	32.13	21.91	96.35	49.92	0.100	0.121	0.102	4,662,862
Prev. + Basal insulin rate	44.34	32.20	22.18	95.95	50.85	0.105	0.124	0.103	3,487,822
Prev. + Carbohydrates	41.66	30.27	20.91	96.56	52.79	0.098	0.115	0.099	5,757,020
**Prev. + Bolus insulin**	**39.67**	**29.29**	**19.93**	**97.26**	**53.15**	0.096	**0.112**	**0.097**	4,777,105

**Prev**.: variables included in the preceding experiment.

**Table 4:** Comparison of this work against relevant studies of the state of the art in glucose forecasting. The best case for each metric is indicated in **bold font**. * Computed using the whole predicted sequence.

AI-Based Model	WARIFA Dataset					
	RMSE (mg/dL)	ParkesAB (%)	ISOZone (%)	Personalization	Interpretability Analysis	Uncertainty Quantification
Stacked-LSTM [39]	38.44	97.77	56.09	Yes	No	No
**Proposed TFT model**	**19.78**	**99.54**	**85.13**	**Yes**	**Yes**	**Yes**
	**OhioT1DM dataset**					
TFT Zhu et al. [37]	32.3 *	n/a	n/a	No	Yes	No
GluNet Li et al. [13]	31.28 *	n/a	n/a	No	No	No
**Proposed TFT model**	27.02 * (39.67)	**97.26**	**53.15**	**Yes**	**Yes**	**Yes**

* Computed using the full predicted sequence; **n/a**: not available.

## Data Availability

The WARIFA dataset presented in this article might be available upon reasonable request. Requests to access the datasets should be directed to Himar Fabelo. The OhioT1DM dataset is available upon reasonable request to the authors.

## Supplementary Materials

The following supporting information can be downloaded at https://www.mdpi.com/article/10.3390/s25154647/s1. Figure S1: Scheme representing the trimester-wise data partition: 60% for the training set, 20% for the validation set, and 20% for the test set. Figure S2: Illustration of the common time grid harmonization between different diabetes-related continuous variables. Table S1: Training, validation and test sets for T1DM Ohio and WARIFA datasets. Table S2: TFT hyperparameters’ explored ranges. Equation (S1): RMSE; Equation (S2): MAE; Equation (S3): MAPE.
