# Supplementary material for: Incorporating Uncertainty Estimation and Interpretability in Personalized Glucose Prediction Using the Temporal Fusion Transformer
Source: Sensors (Basel). 2025 Jul 26;25(15):4647. doi: 10.3390/s25154647 (PMC12349322; doi:10.3390/s25154647)
Supplement: Supplementary file 1 [file sensors-25-04647-s001.zip › sensors-3729848-supplementary.pdf]

Using the Temporal Fusion Transformer to Incorporate Uncertainty Estimation and Interpretability to Personalized Glucose Prediction

Supplementary Material

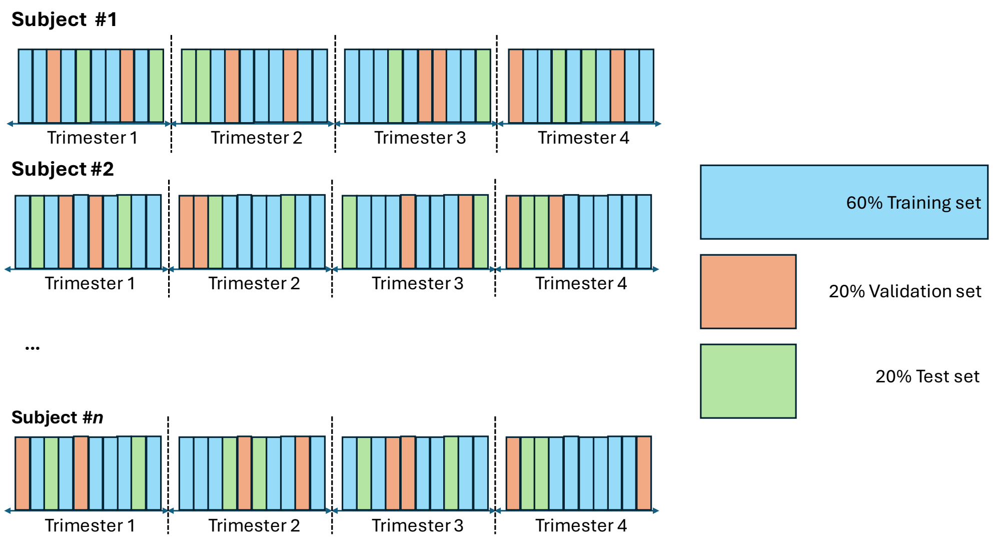

Figure S1. Scheme representing the trimester-wise data partition: 60% for the training set, 20% for the validation set, and 20% for the test set.

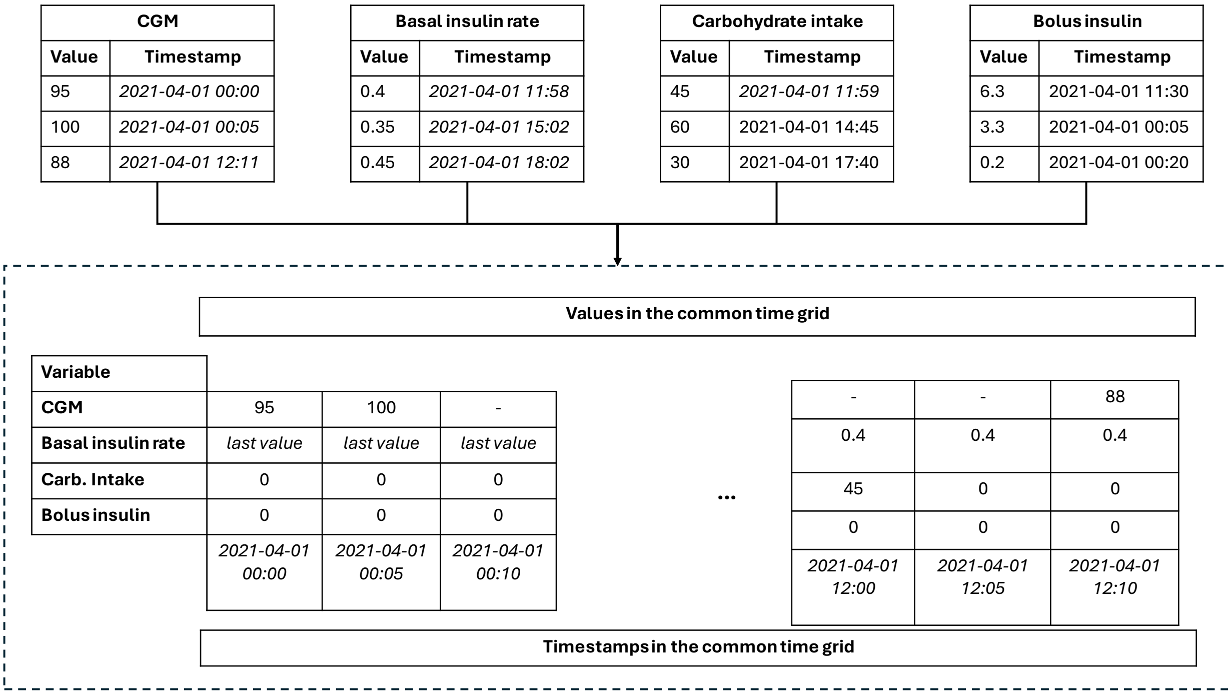

Figure S2. Illustration of the common time grid harmonization between different diabetes-related continuous variables.

Table S1. Training, validation and test sets for T1DM Ohio and WARIFA datasets.

**Data subset**	**OhioT1DM**	**WARIFA**
**Training instances**	74,121	269,688
**Validation instances**	18,530	89,864
**Test instances**	21,020	90,004

Table 2. TFT hyperparameters' explored ranges.

TFT Hyperparameter	Explored range
Number of attention heads	[1, 8]
Hidden size	[16, 265]
Hidden size to process continuous variables.	[8, 128]
Maximum gradient norm	[0.01, 100]
Learning rate	[0.0001, 0.01]
Dropout rate	[0.1, 0.3]

$$RMSE = \sqrt{\frac{1}{N}\sum_{i=1}^{N}(\hat{y}_i - y_i)^2} \quad (1)$$

$$MAE = \frac{1}{N}\sum_{i=1}^{N}|\hat{y}_i - y_i| \quad (2)$$

$$MAPE = \frac{1}{N}\sum_{i=1}^{N}\frac{|\hat{y}_i - y_i|}{y_i} * 100\ \% \quad (3)$$

N being is the number of considered values, $\hat{y}_i$ is the predicted value corresponding to the 50th predicted percentile, and y_i represents the golden reference.
